# Transcriptome analysis for phosphorus starvation-induced lipid accumulation in *Scenedesmus* sp

**DOI:** 10.1038/s41598-018-34650-x

**Published:** 2018-11-06

**Authors:** Fangfang Yang, Wenzhou Xiang, Tao Li, Lijuan Long

**Affiliations:** Key Laboratory of Tropical Marine Bio-resources and Ecology, South China Sea Institute of Oceanology, Chinese Academy of Sciences, Guangzhou, 510301 China

## Abstract

Phosphorus stress was applied to enhance the lipid production in *Scenedesmus* sp. The highest lipid production (350 mg L^−1^) and lipid content (approximately 41.0% of dry weight) were obtained by addition of 2 mg L^−1^ NaH_2_PO_4_·2H_2_O every 2 days, which were higher than those in replete phosphorus. Correspondingly, carbohydrate content decreased significantly. We speculated that phosphorus limitation could block starch biosynthesis, and photosynthate flow tended to fatty acid biosynthesis to cope with stress. To investigate the mechanism that phosphorus stress triggers the carbon fixation to lipid biosynthesis, the transcriptome analysis was carried out by the Illumina RNA-seq platform. A total of 2897 genes were identified as differentially expressed genes. The observed overexpression of lipid production under phosphorus stress was bolstered by up-regulation of genes encoding for DGAT and pyruvate kinase, activation of carbohydrate metabolism pathway and fatty acid biosynthesis, and repression of carbohydrate synthesis-presumably to shunt the carbon flux toward TAG biosynthesis. The transcriptome will be useful to understand the lipid metabolism pathway and obtain the engineering economic algae species aimed at biodiesel production.

## Introduction

It is known that microalgae contain three kinds of major storage products: protein, starch and lipids. Generally, many microalgae store starch as their primary carbon storage compound, whereas switch photosynthetic carbon partitioning toward energy-rich storage compounds such as lipids under environmental stress^1,2^. It might be because the pathways of lipids and starch synthesis compete for common bio-synthetic precursors^3^. Tricarboxylic acid (TCA) cycle metabolites can decline, and then acetyl-CoA is shunted toward fatty acid biosynthesis under stress, resulting that storage lipids increase^4,5^. Therefore, eliminating polysaccharides (e.g., starch) biosynthesis would be a feasible strategy to improve lipid content in the perspective of biodiesel production. Overproduction of lipids was observed in *Chlamydomonas reinhardtii* when starch synthesis was disrupted^6,7^. Ho *et al*.^8^ found that salinity stress switched carbon partitioning from starch to lipid synthesis as a regulator, resulting in an increase of lipid content. However, lipid accumulation accompanied by an increase in starch was observed in several algal species such as *Chlorella zofingiensis*, *Phaeodactylum tricornutum*^9^. So far, the molecular mechanism underlying such a metabolic switching in microalgae remains poorly understood.

Phosphorus, one of the most important nutrients, plays a significant role in cell growth and metabolic processes, especially energy transfer, signal transduction, macromolecule biosynthesis and photosynthesis^10^. Phosphorus limitation is an efficient environmental pressure to induce lipid accumulation. A recent study revealed that low phosphorus resulted in an increase of lipid content and productivity. Meanwhile, carbohydrate content decreased^11^. Additionally, phosphorus stress could modulate lipid classes that a drastic reduction in membrane phospholipids and replacement of these compounds by nonphosphorus glycolipids and sulfolipids were observed^11^. Similar results were also reported in *Dunaliella parva*, which accumulated lipids rather than carbohydrate under low phosphorus^12^. These results have suggested that phosphorus stress could eliminate starch and/or protein biosynthesis, resulting in an increase of the carbon flux toward lipids. However, the signaling pathways by which phosphorus stress triggers lipid accumulation, and how microalgae respond to phosphorus stress at molecular level are still poorly understood. Moreover, lipid content and biomass productivity appear to be in contradiction with each other. Nutrients limitation stimulated lipid accumulation but at the expense of growth for several microalgae^13,14^. *Scenedesmus* sp. increased the cellular lipid content, which in turn decreased the overall lipid production under phosphorus starvation^14^. How to effectively enhance the lipid content under the growth rate being unchanged or improved is an urgent problem to solve.

In this study, a green microalga *Scenedesmus* sp., which was isolated from an outdoor cyanobacterium *Plectonema* sp. culture with a highly alkaline environment, was selected^15^. The alga could grow well in an outdoor raceway system of up to 66 m^3^ in previous experiment, thus, it is meaningful to investigate the alga. According to our early study, phosphorus starvation could induce lipid accumulation, however, the biomass decreased in this alga. To enhance lipid production, the effects of low phosphors-addition feeding intervals on *Scenedesmus* sp. were investigated. Following that, the carbohydrate and protein content were also determined. Furthermore, genes encoding key enzymes in lipid synthesis and other relevant metabolic pathways were illustrated by the Illumina RNA-seq platform. A comprehensive analysis of the gene expression of these pathways in lipid-producing and starch-producing was carried out to understand the regulation mechanism.

## Results and Discussion

### Physiological responses to phosphorus stress

#### Effects of phosphorus concentrations on Scenedesmus sp

**Figure 1:**
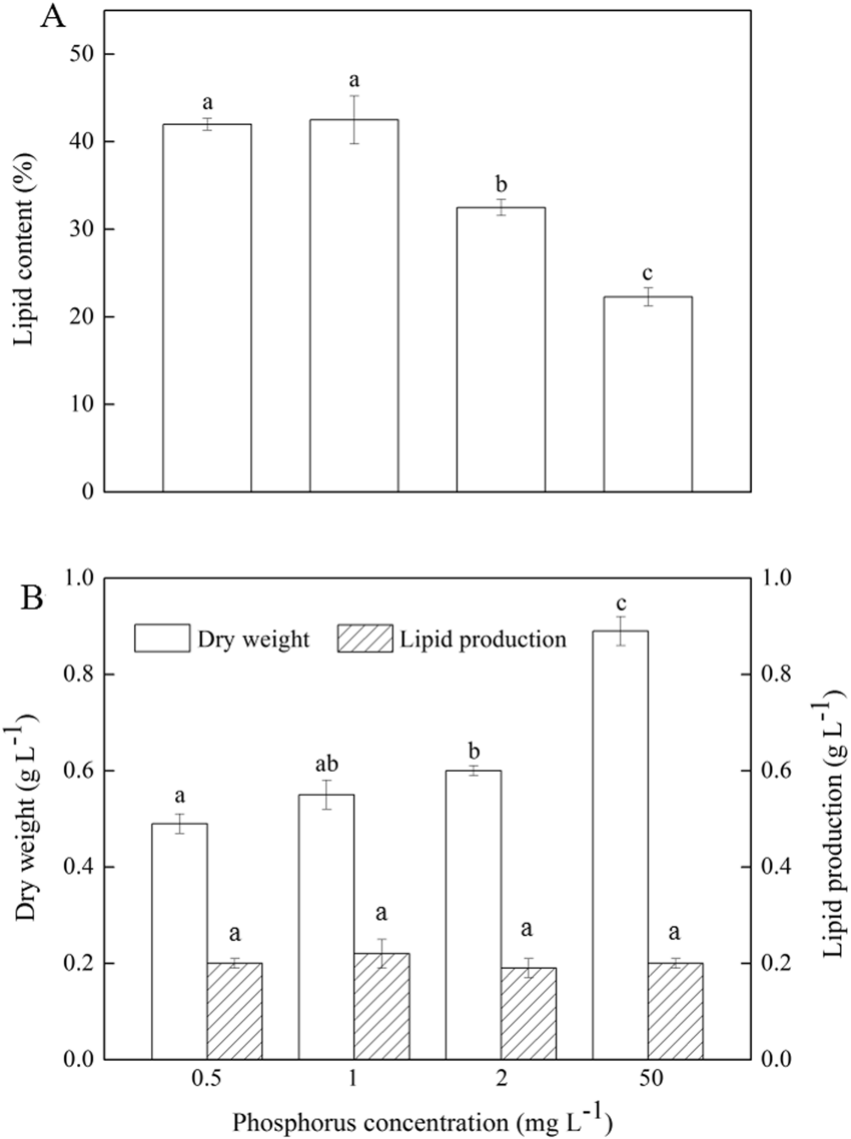
The effects of phosphorus concentrations on (**A**) lipid content; (**B**) growth and lipid production in *Scenedesmus* sp. ^abc^Different letters mean significant differences among groups (*P* < 0.05).

**Figure 2:**
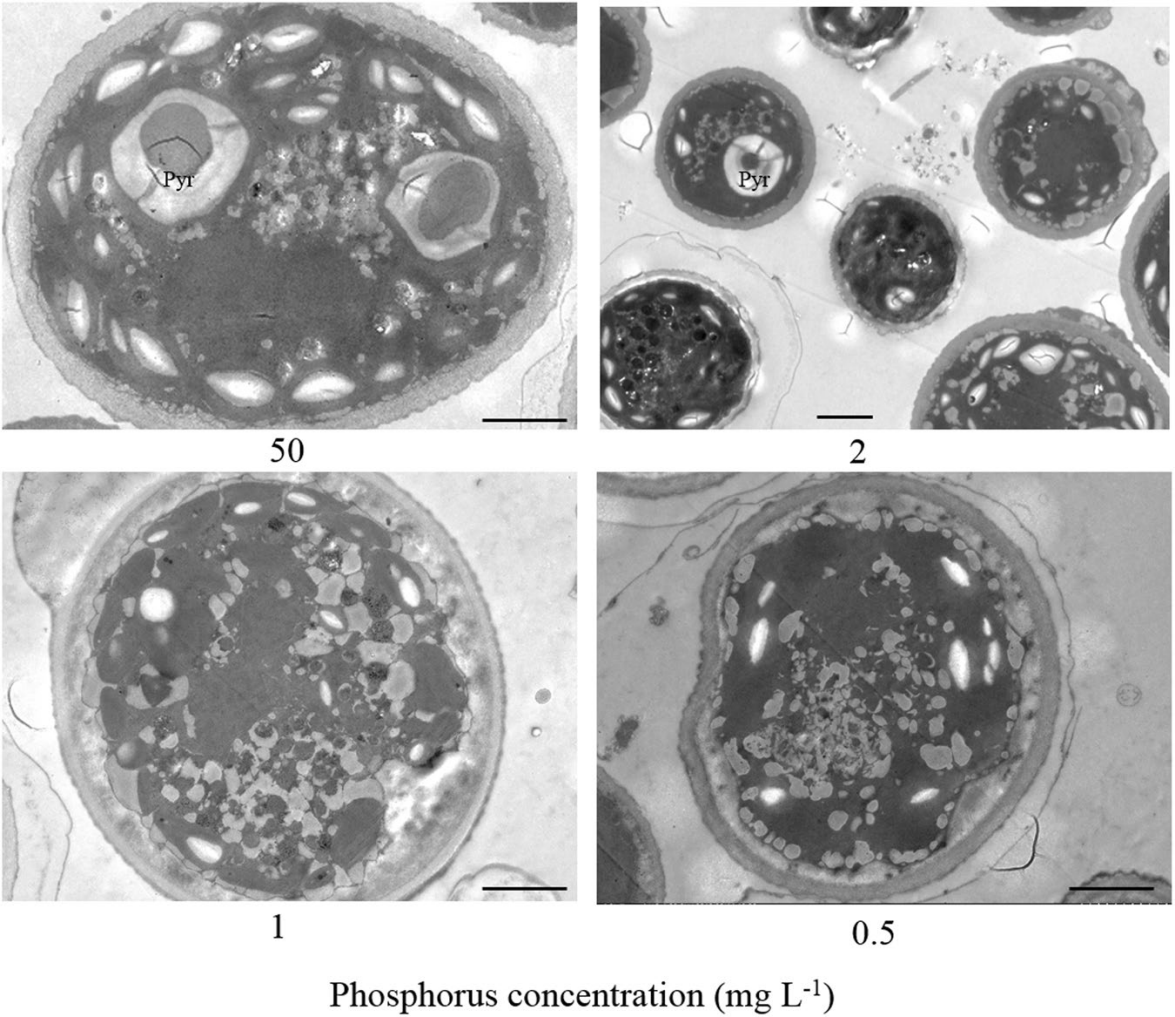
The structure of pyrenoids (Pyr) under different phosphorus concentrations (scale bar = 2 μm).

The accumulation of microalgal lipids is species-specific and largely dependent on cultivation conditions. Phosphorus is viewed as an important factor in lipid synthesis which is mainly incorporated into nucleic and phospholipids, however, only a few studies that phosphorus starvation induces lipid accumulation in microalgae have been carried out^16,17^. In this study, the effects of phosphorus concentrations on *Scenedesmus* sp. were investigated. As illustrated in Fig. 1A, there were remarkable differences in lipid content among various concentrations of phosphorus. Overall, the lipid content increased with a decrease of phosphorus concentrations. The alga growing in 50 mg L^−1^ phosphorus achieved a lipid content of only 22.3%, whereas in 1 mg L^−1^ phosphorus reached a yield of 42.5%. When phosphorus concentration further decreased to 0.5 mg L^−1^, no significant change was observed in lipid content (42.0%). Interestingly, one or several large oval pyrenoids, containing mainly starch and proteins, were observed in algal cells at 50 mg L^−1^ phosphorus, however, the pyrenoids gradually became smaller with a decrease of phosphorus concentration (Fig. 2). Pyrenoids were occasionally observed in individual cells at 2 mg L^−1^ phosphorus. When phosphorus concentration further decreased to 1 mg L^−1^ or 0.5 mg L^−1^, pyrenoids almost disappeared. The results of pyrenoids were consistent with those of lipid content, thus, we speculated that there might be a close link between lipid content and pyrenoids under phosphorus stress. Maybe phosphorus stress can improve lipid content by blocking the starch biosynthesis. But unfortunately, algal biomass decreased from 0.9 g L^−1^ to 0.6 g L^−1^ when phosphorus decreased from 50 mg L^−1^ to 2 mg L^−1^, which led to little improvement in lipid production (Fig. 1B). When phosphorus concentration further decreased to 0 mg L^−1^, the alga almost stop growing. How to effectively enhance the lipid content under the growth rate being unchanged or improved is a challenge. Adjusting the phosphorus feeding interval time could be an effective approach to resolve the contradiction between lipid accumulation and growth.

#### Effects of phosphorus feeding interval time on growth and lipid accumulation

**Figure 3:**
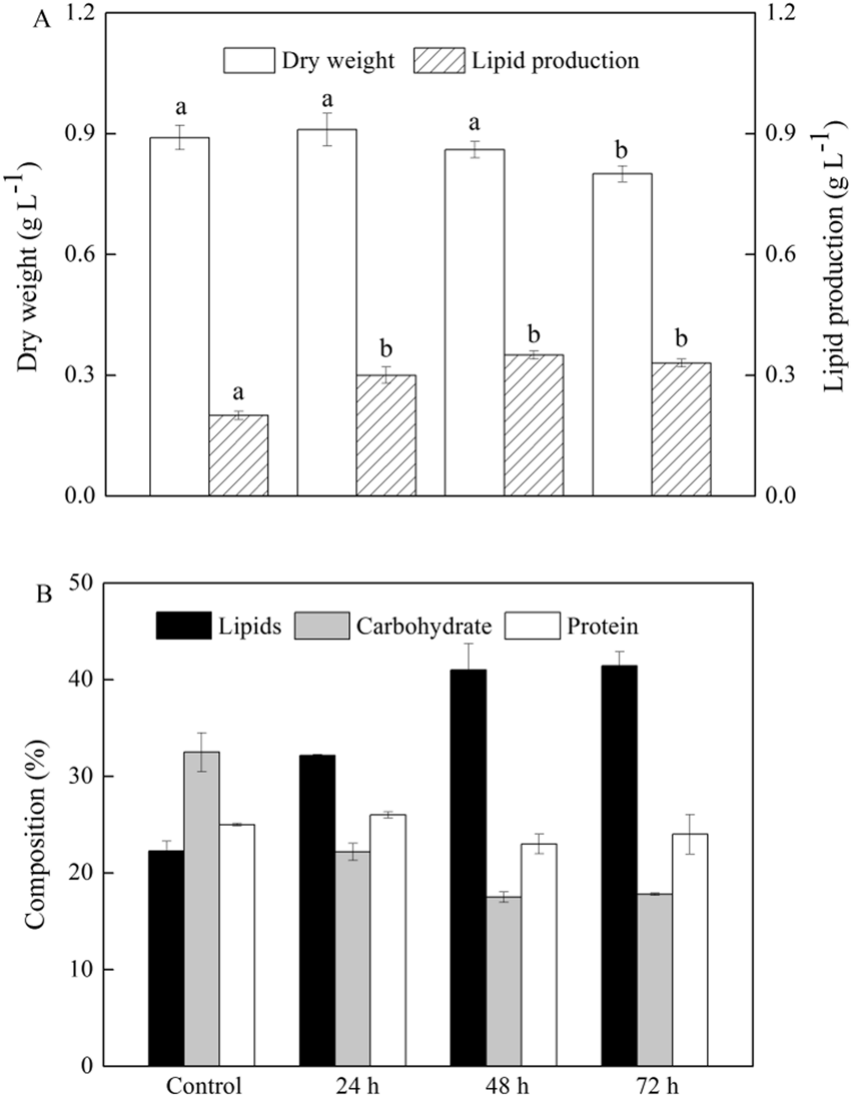
(**A**) The growth and lipid production under phosphorus feeding intervals of 24 h, 48 h and 72 h. (**B**) Cellular biochemical composition of *Scenedesmus* sp. under phosphorus feeding intervals of 24 h, 48 h and 72 h. (black bar) lipids; (gray bar) carbohydrate; (white bar) protein. ^ab^Different letters mean significant differences between groups (*P* < 0.05).

To investigate an optimal time interval for cell growth and lipid accumulation of *Scenedesmus* sp., phosphorus feeding intervals of 24 h, 48 h and 72 h were tested with 2 mg L^−1^ NaH_2_PO_4_·2H_2_O. As shown in Fig. 3A, dry weight was the highest when phosphorus was supplied at an interval of 24 h, which was comparable to that obtained by the control group (50 mg L^−1^ NaH_2_PO_4_·2H_2_O). With the prolonging the feeding time interval of phosphorus (48 h and 72 h), dry weight gradually decreased. At an interval of 72 h, dry weight was significantly lower than that obtained by the control group. By contrast, the lipid content of 48 h interval treatment reached to 41.0%, which was significantly higher than that of 24 h interval treatment and control group, as shown in Fig. 3B. When feeding time interval of phosphorus was prolonged to 72 h, no significant difference was observed in lipid content. As a result, the lipid production reached the maximum value of 0.35 mg L^−1^ at 48 h interval treatment due to higher biomass and lipid content, which was 84.2% higher than that obtained by control group (Fig. 3A,B). Considering the lipid production and cost, 48 h interval treatment was chosen as the optimal time interval of phosphorus feeding. Under the optimal conditions, the lipid profiles and fatty acid composition of *Scenedesmus* sp. were studied. When phosphorus was supplied at an interval of 48 h, the content of neutral lipid (approximately 80% of total lipids) was slightly higher than that obtained by control group while the proportion of phospholipid was reduced from 6.1% to 4.3% of total lipids (Supplementary Fig. S1A). Additionally, no significant differences in fatty acid composition were observed, however, the exact amount of some fatty acids altered (Supplementary Fig. S1B). At an interval of 48 h, C18:2 and C18:3 were significantly lower while C16:0 and C18:1 increased compared to the control group. The contents of saturated fatty acids increased from 35.9% to 38.7%, which suggested that *Scenedesmus* sp. could tend to produce saturated fatty acids during phosphorus limitation.

Previous studies have demonstrated that algal cells tend to accumulate lipids under nutrients-depletion^18^. N-starvation is the most widely explored strategy for the enhancement of lipid accumulation. However, the inverse relation between lipid accumulation and cell growth has been recorded in many microalgae. Compared with nitrogen and sulfur deprivation, limited phosphorus addition could support continuous cell growth as well as enhance lipid accumulation, which seems more effective in inducing lipid accumulation. These results are similar to previous reports, where lipid storage increased by phosphorus deficiency^16,17^. The reason could be that the available phosphorus is utilized for synthesis of enzymes and essential cell structures under phosphorus deficiency, any carbon dioxide subsequently fixed is therefore converted into lipids or protein rather than carbohydrate. But the exact mechanism is not very clear and needs more investigations.

It is speculated that microalgae can switch photosynthesis carbon partitioning toward energy-rich storage macromolecules mainly lipids under stress. *Pseudochlorococcum* used starch as a primary energy storage product, however, cells shifted the carbon partitioning into lipids as a secondary storage product after N-depletion^19^. *Haematococcus pluvialis* could produce carbohydrates intensively, and later convert to fatty acids in response to nutrient deprivation^20^. Similarly, a convertible relationship between carbohydrate and lipids has been observed in *Chlamydomonas reinhardtii*^6^. Therefore, it is important to measure the change in these macromolecules for biofuel production. In this study, cellular biochemical compositions including carbohydrate and protein were analyzed. Compared to control group, the carbohydrate content all significantly decreased at different phosphorus feeding interval time, as shown in Fig. 3B. The carbohydrate content gradually decreased from 32.5% (control group) to 22.2% at 24 h interval, which were consistent with the results of pyrenoids. The carbohydrate content further decreased at 48 h interval. But, when the feeding interval time was added to 72 h, carbohydrate content tended to remain constant. By contrary, no significant differences were observed in protein content among different time interval of phosphorus feeding. These results indicated that total lipid content and yield were accelerated, correspondingly the carbohydrate content and yield decreased under phosphorus stress. Therefore, we speculated that carbon flux transferred into lipid biosynthesis from starch when alga was faced with phosphorus stress.

Morphologically, one or several large oval pyrenoids appeared in algal cells at 50 mg L^−1^ phosphorus. However, pyrenoids were only observed in some cells at an interval of 24 h, and disappeared in the cells at an interval of 48 h and 72 h. Taken together, the results of pyrenoids were consistent with the changes of cellular biochemical composition, which further supported our speculation that carbon flux transferred into lipid biosynthesis from starch under phosphorus stress. These results extend the idea of blocking starch synthesis for improvement of lipid production in *Scenedesmus* sp. although the link between lipid accumulation and pyrenoids under phosphorus stress require further investigation.

### Transcriptomeh analysis of *Scenedesmus* sp. response to phosphorus stress

#### Illumina sequencing and assembly

Although this phenomenon that phosphorus starvation can trigger lipid accumulation for several microalgae is well-known, the signaling pathways and molecular mechanism are still poorly understood. *Scenedesmus* sp. may be a candidate alga for biodiesel production since it can grow well in an outdoor raceway system and has a high lipid content by phosphorus starvation. The transcriptome analysis might be contribute to understand the lipid metabolism process. Furthermore, it might provide promising approaches to further promote lipid accumulation by genetic manipulations. In the future, a desired strain for biodiesel production by inhibition of over-expression of the related genes identified here may be obtained. In this study, the transcriptome analysis was carried out in P-starvation and P-replete conditions. As shown in Table 1, the average quality value was ≥20 for 98% of the cycle with a near zero ambiguous “N”. The GC contents were 52.75% and 52.60% for P-replete and P-starvation, respectively, which suggested that the sequencing was highly accurate and reliable. By sequencing, P-replete group produced 28108116 raw reads and 26517528 clean reads, while P-starvation group produced 29121522 raw reads and 27504706 clean reads. Then the clean reads were assembled using Trinity software to remove adaptor sequences and the exclusion of contaminated or short reads. 78807 contigs with a mean length of 457nt and 46869 unigenes with a mean length of 891 nt were obtained in P-replete group. Similarly, the reads in P-starvation group were assembled into 80446 contigs with a mean length of 445 nt and 47682 unigenes with a mean length of 875 nt.

**Table 1:** Summary of output statistics by Illumina sequencing.

Samples	Raw Reads	Clean Reads	Total Clean Nucleotides (nt)	Q20 (%)	N (%)	GC (%)
P-replete	28,108,116	26,517,528	2,386,577,520	97.79%	0.01%	52.75%
P-starvation	29,121,522	27,504,706	2,475,423,540	97.79%	0.01%	52.60%

Among 39975 identified unigenes, 20983 unigenes were successfully annotated using the NR, Swiss-Prot, GO, KEGG and COG database (Supplementary Table S1). Specifically, the E-value distribution showed that 36.3% of the mapped sequences had strong homology with the E-value < 1.0E^−45^, whereas remaining 63.6% of the homolog sequences ranged from 1.0E^−5^ to 1.0E^−45^ (Supplementary Fig. S2A). The distribution of similarity values showed that there was a low similarity, suggested that the alga might be a new species unpublished. So far, the molecular information of *Scenedesmus* sp. has been very limited; no genomic sequences are available. Only 2.5% of the sequences had hits with similarity higher than 80%, while 97.5% of unigenes showed similarity of ranging from 17% to 80% (Supplementary Fig. S2B). In terms of species distribution, the majority of the annotated sequences corresponded to known nucleotide sequences of microalgae species, with 30.0% of the sequences showing the highest homology to sequences from *Volvox carteri f. nagariensis*, followed by *Chlorella* sp. (25.6%) and *Chalmydomonas reinhardtii* (23.0%) (Supplementary Fig. S2C). The little proportion of unigenes belonging to *Scenedesmus* sp. might be the results of searching in public databases which contained few data of *Scenedesmus* sp. Overall, the species with the most BLAST unigenes belonged to Chlorophyta.

GO-annotated consensus sequences were categorized into about 44 functional groups, including biological process, cellular component, and molecular function (Supplementary Fig. S3). The effectiveness of the annotation process and the completeness of the transcriptome library were further evaluated by COG classification (Supplementary Fig. S4). COG-annotated putative proteins were classified functionally into at least 25 molecular families. Among these COG categories, the cluster for “General function prediction only” represented the largest group, followed by “Cell wall”, “Replication, recombination and repair”, and “Carbohydrate transport and metabolism”. The smallest groups were “Nuclear structure”, “RNA processing and modification”, and “Extracellular structures”.

#### Differentially expressed genes analysis

The gene expression in response to phosphorus stress was determined by IIIumina RNA sequencing technology. A total of 2897 genes were identified as the differentially expressed genes, out of which 1853 and 1044 genes were respectively up- and down-regulated by the phosphorus stress. Most of the differentially expressed genes are hypothetical genes, which indicated how little is known about gene function in microalgae. In the GO classification analysis, differentially expressed genes were assigned to three main GO functional categories including biological process, cellular component, and molecular function (Supplementary Fig. S5). The largest percentage of sub-category in the “biological process” category was metabolic process, followed by cellular process, signaling-organism process and response to stimulus. The largest percentage of sub-category in the “molecular function” category was catalytic activity, followed by binding and transporter activity. These results could be contribute to understand the molecular mechanism for phosphorus stress-induced lipid accumulation. The levels of differentially expressed genes were analyzed by a metabolic pathway map and KEGG category. Under P-stressed condition, genes associated with lipid biosynthetic process, TAG biosynthesis and carbohydrate metabolism pathway had significant differences of transcripts compared to the P-replete condition; reflecting higher lipid content was observed in P-starvation. 66 differentially expressed genes were identified in lipid metabolism pathway, while 142 differentially expressed genes were detected in carbohydrate metabolism pathway.

**Figure 4:**
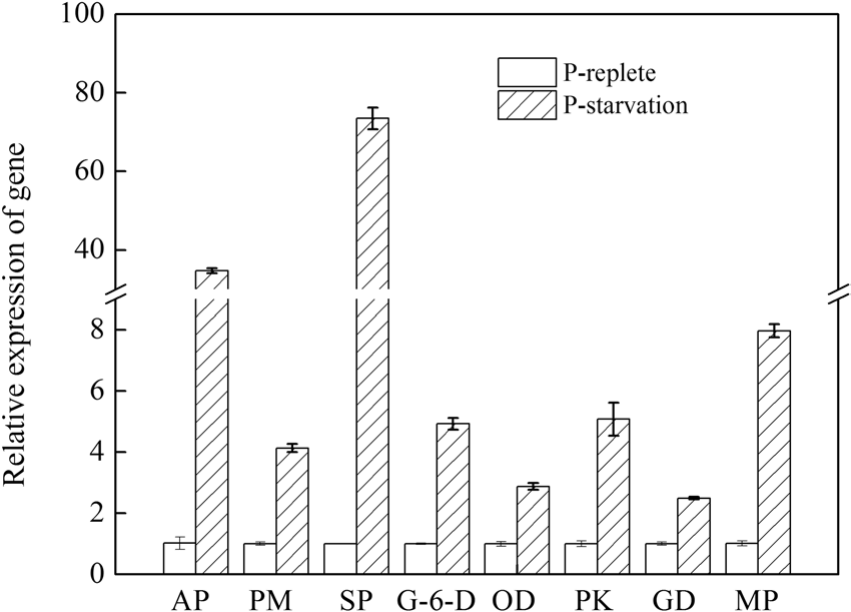
QPCR analysis of the selected differentially expressed genes. AP: Acid phosphatase; PM: Phosphoglycerate mutase; SP: Sucrose phosphatase; G-6-D: Glucose-6-phosphate dehydrogenase; OD: Oxidoreductase; PK: Pyruvate kinase; GD: Glutamate dehydrogenase; MP: Metallo-dependent phosphatase.

To verify the results of transcriptome sequencing, nine unigenes were selected for qRT-PCR analysis (Fig. 4). The results showed that the expression of all nine genes was consistent between the qRT-PCR and transcriptome analyses. These results indicated that the sequencing method was effective.

#### Identification of differentially expressed genes involved in fatty acid biosynthesis

**Figure 5:**
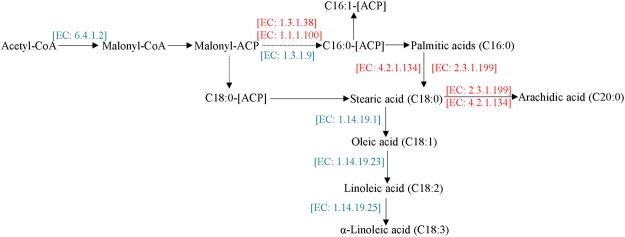
The expression levels of genes involved in the fatty acid biosynthesis. Red genes are up-regulated genes, while the green genes are down-regulated genes.

The abundances of differentially expressed genes in the lipid metabolism pathway were shown in Supplementary Fig. S6. Overall, the majority of genes governing *de novo* fatty acid biosynthesis were identified as being overexpressed in phosphorus-limited cells, as shown in the metabolic pathway level and fatty acid biosynthesis module (Fig. 5). The first committed step in fatty acid synthesis is the transduction of acetyl-CoA into malonyl-CoA, which is catalyzed by acetyl-CoA carboxylase (ACCase, EC: 6.4.1.2 6.4.1.3). ACCase is considered a key enzyme for fatty acid synthesis, which generally contain two forms of ACCase: homomeric, and heteromeric ACCase which has four subunits. Previous study shown that lipid accumulation could be associated with the expression levels of accD encoding heteromeric ACCase, and not with gene encoding homomeric ACCase^21^. Roesler *et al*.^22^ revealed that lipid content wasn’t significantly promoted when ACCase gene was transferred into algal cells. In this study, one gene encoding ACCase was significantly down-regulated while no significant changes in the expression of other genes encoding ACCase were observed in response to P-starvation. We speculated that the expression level of the gene was likely to be little correlation with fatty acid synthesis. Next, malonyl-CoA acyl-carrier protein transacylase (EC: 2.3.1.39) catalyzes malonyl-CoA to ACP. Then a series of acylcondensation, reduction, dehydration, and again reduction reactions occur, which are catalyzed by the fatty acid synthase (FAS). The synthesis ceases after six or seven cycles when the number of carbon atom reaches sixteen (C16:0-[ACP]) or eighteen (C18:0-[ACP]). The end products of fatty acid synthesis, mainly palmitic and stearic acids, are generated after cleaving off ACP residues. The expression of one unigene encoded enoyl-ACP reductase (EAR, EC: 1.3.1.9 1.3.1.10) was repressed, whereas four encoded 3-oxoacyl-ACP reductase (KAR, EC: 1.1.1.100) were up-regulated in P-stressed cells. These results suggested that KAR could be key factor in lipid accumulation, instead of ACCase.

In the fatty acid elongation process, four unigenes were all up-regulated under phosphorus stress, including one encoded mitochondrial trans-2-enoyl-CoA reductase (EC:1.3.1.38), two encoded 3-ketoacyl-CoA synthase (KCS, EC: 2.3.1.199), and one encoded very-long-chain (3 R)-3-hydroxyacyl-CoA dehydratas (PHS1, EC: 4.2.1.134), respectively (Fig. 5). These results suggested that *Scenedesmus* sp. could tend to produce long chain fatty acids under phosphorus stress.

Additionally, fatty acid desaturation contains: (1) the formation of monounsaturated fatty acids from saturated fatty acids; (2) the formation of polyunsaturated fatty acids. Ten unigenes were identified, of which only three unigenes were significantly repressed in biosynthesis of unsaturated fatty acids in the P-starvation case. Three unigenes encoded acyl-lipid omega-3 desaturase (EC: 1.14.19.25 1.14.19.35 1.14.19.36) converting C18:2 to C18:3, delta-9 desaturase (EC: 1.14.19.1) converting C18:0 to C18:1, and delta-12 desaturase (EC: 1.14.19.23 1.14.19.45) catalyzing the formation of C18:2 from C18:1, respectively. Other unigenes encoding 3-oxoacyl-ACP reductase (KAR, EC: 1.1.1.100), acyl-CoA oxidase (EC: 1.3.3.6), and very-long-chain (3 R)-3-hydroxyacyl-CoA dehydrates (PHS1, EC: 4.2.1.134) adding two carbon on fatty acids were up-regulated. These results suggested that *Scenedesmus* sp. could tend to produce saturated fatty acids during phosphorus limitation, which were consistent with the changes in fatty acids.

#### Identification of differentially expressed genes involved in TAG biosynthesis

TAG biosynthesis occurs in endoplasmic reticulum (ER) using the fatty acid and glycerol as substrates in microalgae^23^. Glycerol was firstly catalyzed by glycerol kinase (GK, EC: 2.7.1.30) to form glycerol-3-phosphate (G3P). Then glycerol-3-phosphate acyltransferase (GPAT, EC: 2.3.1.15) catalyzed G3P to form 1-Acyl-sn-glycerol-3-phosphate, which was further converted into 1,2-Diacyl-sn-glycerol-3-phosphate by 1-Acyl-sn-glycerol-3-phosphate acyltransfe- rase (AGPAT, EC: 2.3.1.51). Additionally, 1,2-Diacyl-sn-glycerol-3-phosphate was also formed from phosphatidylcholine by phospholipase. After acylation, phosphatidate phosphatase (PP, EC: 3.1.3.4) catalyzed 1,2-Diacyl-sn-glycerol-3- phosphate to form 1,2-sn-Diacyl-glycerol (DAG), which acted as a precursor for many compounds, such as TAG, 1,2-diacyl-3-beta-galactosyl-sn-glycerol (SQDG), or phosphatidylethanolamine (PE). DAG was then catalyzed to TAG by several kinds of enzymes, among which phospholipid: diacylglycerol acyltransferase (DGAT, EC: 2.3.1.20) was a crucial and rate-limiting enzyme catalyzing the last step in the TAG biosynthesis^24,25^. DGAT is thought to be one of the most promising target genes for genetic engineering to enhance TAG content. In this study, one gene encoded DGAT was up-regulated under phosphate stress, increasing the flux of carbon toward TAG. These observations suggested that DGAT might play an important role during the period of maximal TAG accumulation in *Scenedesmus* sp. Previous studies also demonstrated that over-expression of DGAT could improve the lipid content in *Chlamydomonas reinhardtii*, *Tetraselmis chui* and *Neochloris oleoabundans*^25–27^. However, the detail function of DGAT in *Scenedesmus* sp. is worthy of further investigation. Finally, oil bodies formed by the combination of oil proteins with TAG were released from the ER into the cytoplasm^28^.

#### Identification of differentially expressed genes involved in carbohydrate biosynthesis

Lipids and starch could be interconvertible, since starch synthesis shared common precursors with lipid synthesis. In previous study, one or several large oval pyrenoids were observed in algal cells at 50 mg L^−1^ phosphorus. However, pyrenoids could be observed in few cells under phosphate stress, which suggested that lipid accumulation could be tightly connected with disappearance of pyrenoids under phosphate-limited condition. Therefore, the expression levels of genes involved in carbohydrate metabolism pathway were determined. Four unigenes encoded beta-glucosidase (EC: 3.2.1.21), one encoded maltase-glucoamylase (EC: 3.2.1.20 3.2.1.3), one encoded alpha-amylase (EC: 3.2.1.1), and seven encoded beta-fructofuranosidase (EC: 3.2.1.26) were all up-regulated by phosphate stress. Beta-glucosidase, maltase-glucoamylase, alpha-amylase, and beta-fructofuranosidase could convert polysaccharides (Glucoside, maltose, cellulose, sucrose) to glucose, which accelerated carbohydrate metabolism, resulting in a decrease of carbohydrate content and an increase of glucose content. Glucose can be converted into acetyl-CoA, which is the precursor for fatty acid synthesis. So, up-regulation of these genes could be key factors for the enhancement of lipid accumulation. Meanwhile, several down-regulated genes including one encoded starch synthase (EC: 2.4.1.21), one encoded 1,4-alpha-glucan branching enzyme (EC: 2.4.1.18) were observed, which could suppress carbohydrate synthesis. The expression level of gene encoding aldolase (EC: 4.1.2.13) decreased, which could suggested that conversion of G3P to carbohydrate was suppressed. Since G3P was also the precursor for glycerol backbone of TAG, more carbon skeletons flew into acetyl-CoA and TAG biosynthesis under phosphorus limitation. The differential expression of these genes was consistent with a decrease of carbohydrate content. Additionally, the differentially expressed genes encoded lactate dehydrogenase (EC: 1.1.2.4) and pyruvate kinase (EC: 2.7.1.40) were also up-regulated, which stimulated the formation of pyruvate, resulting that carbon source was shunted toward lipid biosynthesis.

#### Distribution of genes in lipids and carbohydrate metabolism pathway

**Figure 6:**
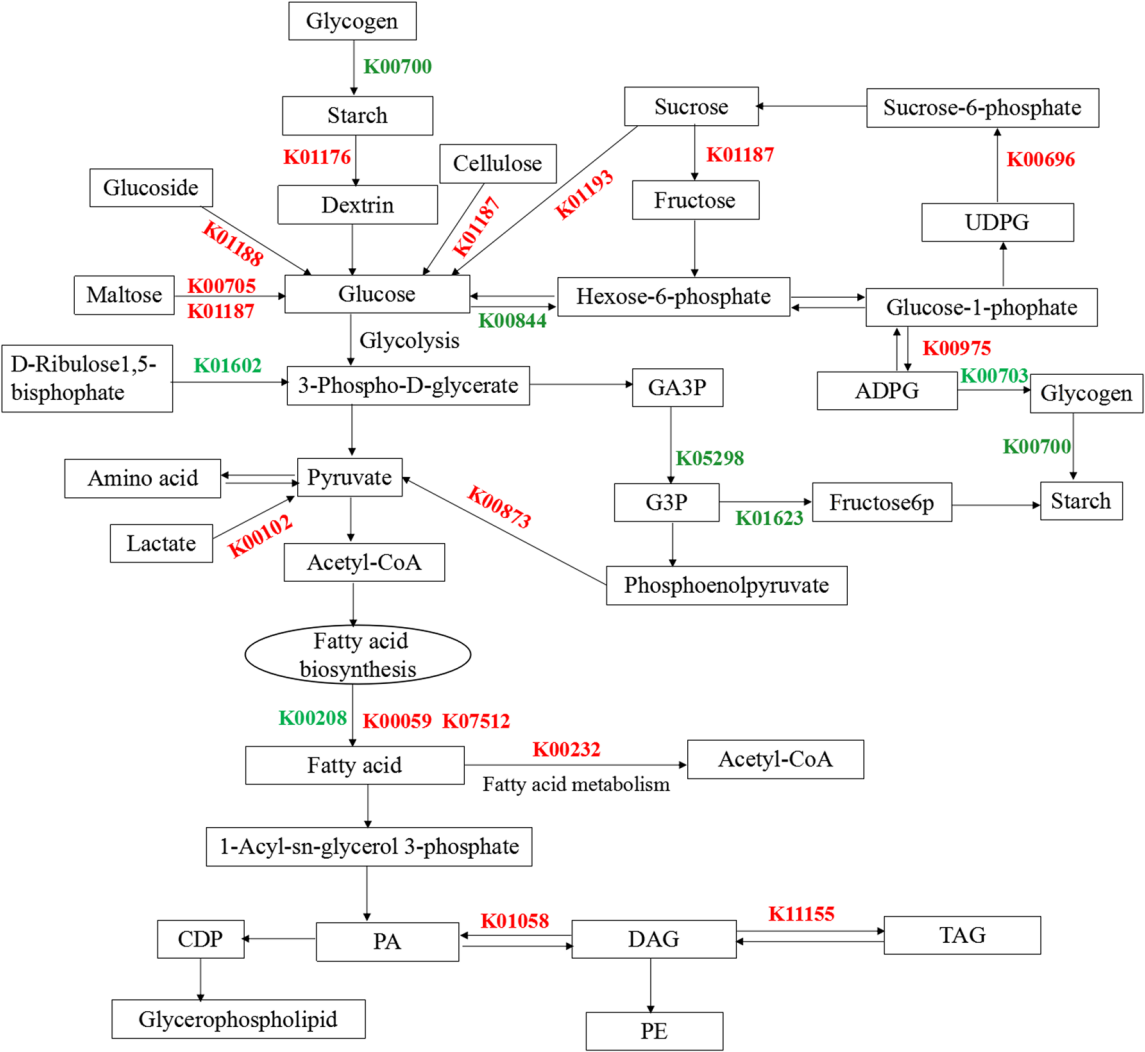
Distribution of differentially expressed genes in the lipid and carbohydrate metabolism pathway. Red genes are up-regulated genes, and the green genes are down-regulated genes. UDPG: Uridine diphosphate glucose; ADPG: Adenosine diphosphate glucose; GA3P: Glyceraldehyde-3-phosphate; G3P:Glycerol-3-phosphate; CDP: Cytidine diphosphate; PA: Palmitic acid; DAG: Diacyl glycerol; TAG: Triacylglycerol; PE: Phosphatidylethanolamine.

**Figure 7:**
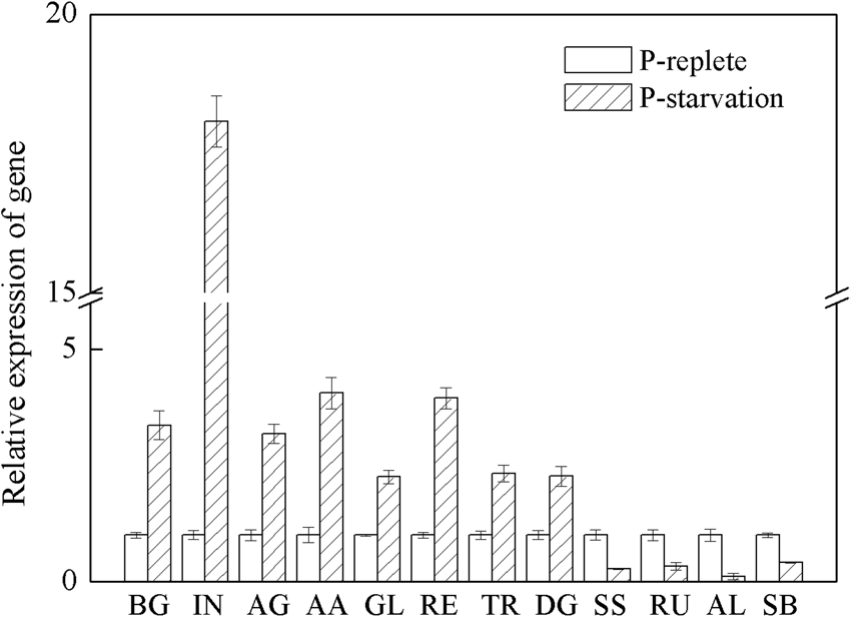
QPCR analysis of differentially expressed genes in lipids and carbohydrate metabolism pathway. BG: Beta-glucosidase; IN: Invertase; AG: alpha-glucosidase; AA: alpha-amylase; GL: Glucanotransferase; RE: Reductase; TR: Trans-2-enoyl-CoA reductase; DG: DGAT; SS: Starch synthase; RU: Rubisco; AL: Aldolase; SB: Starch Branching Enzyme.

Based on these identified enzymes, we reconstructed the fatty acid biosynthesis and carbohydrate metabolism pathway by referencing previous reports, as shown in Fig. 6^29–31^. The relative expression of mainly related differentially expressed genes were list in Fig. 7. The proteins coded by these differentially expressed genes could be found in Table 2. KAS and DGAT, important enzymes in fatty acid synthesis, were significantly up-regulated under phosphorus stress, suggested that KAS and DGAT might be step-limiting enzymes in *Scenedesmus* sp. Additionally, these results of carbohydrate synthesis and metabolism revealed that the source of carbon for the cycle could be shunted toward fatty acid biosynthesis under phosphate stress. Therefore, we speculated that the sink for carbohydrate synthesis, enhancement of carbohydrate metabolites, and improvement of expression levels of *KAS* and *DGAT* resulted that cellular lipids increased. In summary, these data described above might be contribute to understand the lipid metabolism process, although the functional characteristics of some genes associated with biofuel biosynthesis and metabolism (especially genes listed in the Table 2) require further confirmation. Additionally, increasing TAG synthesis or blocking starch synthesis by genetic manipulations could be promising approaches to promote lipid accumulation in microalgae. In the future, we may obtain a desired strain for biodiesel production by inhibition of over-expression of the related genes identified here.

**Table 2:** The differentially expressed genes in the lipid and carbohydrate metabolism pathway.

KEGG Entry	Protein	Expression changes	ECnumber
K01188	Beta-glucosidase	Up	3.2.1.21
K01193	Invertase	Up	3.2.1.26
K01187	alpha-glucosidase	Up	3.2.1.20
K01602	Rubisco -1.60	Down	4.1.1.39
K05298	G3PDH	Down	1.2.1.13
K01623	Aldolase-3.3	Down	4.1.2.13
K00873	Pyruvate kinase	Up	2.7.1.40
K00844	Hexokinase	Down	2.7.1.1
K00975	ADP glucosepyrophosphorylase	Up	2.7.7.27
K00700	Starch Branching Enzyme-1.29	Down	2.4.1.18
K01176	alpha-amylase	Up	3.2.1.1
K00705	Glucanotransferase 1.17	Up	2.4.1.25
K01187	Glucanotransferase	Up	3.2.1.20
K00102	Lactate dehydrogenase	Up	1.1.2.4
K00059	Reductase	Up	1.1.1.100
K00208	Reductase	Down	1.3.1.9 1.3.1.10
K00232	Acyl-CoA oxidase 2.4	Up	1.3.3.6
K07512	Trans-2-enoyl-CoA reductase 1.22	Up	1.3.1.38
K11155	DGAT 1.18	Up	2.3.1.20
K01058	Hydrolases	Up	3.1.1.32
K00696	Sucrose-phosphate synthase 1.35	Up	2.4.1.14
K00703	Starch synthase -1.867	Down	2.4.1.21

## Material and Methods

### Strain and culture conditions

The green microalga *Scenedesmus* sp. was isolated from an outdoor cyanobacterium *Plectonema* sp. culture with a highly alkaline environment. The alga was cultured in a modified soil extract (SE) medium composed of the following components: NaHCO_3_, 2 g L^−1^; NaH_2_PO_4_·2H_2_O, 50 mg L^−1^; NaNO_3_, 0.5 g L^−1^; CaCl_2_, 0.02 g L^−1^; MgSO_4_·7H_2_O, 0.05 g L^−1^; KCl, 0.1 g L^−1^ and the same levels of trace elements as described by Yang *et al*.^15^. The cultures were incubated in 500-mL Erlenmeyer flasks with 300 mL of culture media at 24 ± 1 °C and illuminated by fluorescent lamps for 24 h (120 µmol m^−2^s^−1^).

The algal cells were cultured in the modified SE medium without phosphorus for 6 d to deplete the polyphosphates stored in cell. Then cells were concentrated by centrifugation and resuspended in the modified SE medium with 2 mg L^−1^ NaH_2_PO_4_·2H_2_O. The different phosphorus feeding intervals of 24 h, 48 h and 72 h were tested with 2 mg L^−1^ NaH_2_PO_4_·2H_2_O to investigate an optimal time interval of phosphorus feeding for cell growth and lipid accumulation in *Scenedesmus* sp. After 9 d, the algal suspension were collected to determine the growth rate and lipid content.

### Biomass determination

In all experiments, three series of batch cultures were set up in parallel for each treatment. Algal biomass was measured by dry weight. Aliquots of 20 mL microalgal suspension were filtered by preweighed GF/C filter paper (Whatman, Poole, UK). The filter paper with biomass was then dried at 105 °C to a constant weight. After cooling down to room temperature, the filter paper was weighed.

### Determination of lipids, carbohydrate and protein content

After cultivation for 9 d, cells were harvested by centrifugation (3000 × *g*, 5 min) and dried. Intracellular lipids were extracted, as previously reported by Khozin-Goldberg *et al*.^32^. Briefly, a mixture of methanol-dimethyl sulphoxide, diethyl ether and hexane (1:1:1, v/v/v) were used to extract the lipids. When the algae debris was removed, water was added into the organic solvent loaded with lipids, forming a liquid-liquid separation state. Finally, the upper layer, including diethyl ether and lipids, was transferred into the weighed vial and dried by a stream of N_2_. The extracted lipids were quantified and analyzed. Lipid production was calculated by the following equation: Y = M_t_ × L_t_ (Y, lipid production/g L^−1^; M_t_, biomass concentration/g L^−1^; L_t_, lipid content/% dw).

The protein content was estimated by measuring the total nitrogen content according to the Kjeldahl method^33^. The nitrogen-to-protein conversion factor was 6.25.

Total carbohydrate content was measured by phenol-sulfuric method^34^.

### Lipid analysis and fatty acid composition

Lipid class separation was performed by silica gel column chromatography, according to the method illustrated by Christie^35^. Typically, the samples of lipids re-suspended in chloroform were loaded onto a silica gel column (Agela, Tianjin, China). Neutral lipid, phospholipid and glycolipid were successively eluted using chloroform, acetone and methanol, respectively. Each component was dried by a stream of N_2_ and then weighed.

The fatty acid composition of the lipids was analyzed by GC-MS with an Omegawax 250 polyethylene glycol capillary column using the method reported by Khozin-Glodberg *et al*.^32^.

### Transcriptome analyses

20 mL of algal cells were harvested from the phosphorus-replete (50 mg L^−1^ NaH_2_PO_4_·2H_2_O) and -starved (feeding interval of 48 h with 2 mg L^−1^ NaH_2_PO_4_·2H_2_O) cultures respectively and concentrated by centrifugation (3000 × *g*, 5 min). The concentrated samples were transferred into tubes and frozen in liquid nitrogen. Total RNA was extracted using Trizol reagent following the manufacturer’s instructions, and treated with RNase-free DNase I (TaKaRa) to remove genomic DNA. RNA concentration was determined using NanDrop1000 spectrophotometer (Thermo Scientific, DE). The quality of RNA was evaluated using an Agilent 2100 Bioanalyzer (Agilent Technologies, Palo Alto, CA).

A kit (BGI Tech) was added to the collected RNA to remove rRNA. Then mRNA was disrupted into short fragments by fragmentation buffer. Using the cleaved RNA fragments as templates, random primers were used for the first strand cDNA synthesis. Second strand cDNA synthesis was conducted using buffer, DNA polymerase I, dATPs, dGTPs, dCTPs, dUTPs and RNase H. These cDNA fragments went through an end repair process, and then connected to sequencing adapters. The product was purified with the MiniElute PCR Purification Kit, followed by PCR amplification to create the final cDNA library. Finally, the library was sequenced with an Illumina HiSeq2000 instrument.

### De novo assembly, gene annotation and metabolic pathway analysis

The raw reads were cleaned by discarding adapter sequences, low-quality ends (<QV30), and reads shorter than 50 bp. *De novo* assembly of the clean reads was conducted using Trinity software.

All unigenes were annotated using BLAST with a cutoff E-value of 1e^−5^ based on various protein databases, i.e. Nr (NCBI non-redundant protein sequences), Nt (NCBI non-redundant nucleotide sequences), Swiss-Prot (a manually annotated and reviewed protein sequence database) to analyze the most descriptive annotation of each sequence. Function analysis of all unigenes was performed by subjecting to COG (Cluster of Orthologous Groups of proteins), KEGG (Kyoto Encyclopedia of Genes and Genomes) and GO (Gene ontology).

### Quantification and differential expression analysis

The fragments per kilobase of exon per million fragments (FPKM) was applied to calculate the expression of unigenes according to the method of Audic and Claverie^36^. Statistical analysis of the different cDNA libraries was carried out to compare gene expression under phosphorus stress. A false discovery rate (FDR) of <0.001 and log2 (fold change) of ≥±1 were set as the threshold to evaluate the significance of the gene expression difference, by which the unigenes with significantly differential expression were selected. For pathway enrichment analysis, all differentially expressed genes were annotated to GO and KEGG database.

### Quantitative real-time PCR

Eight differentially expressed genes were randomly selected for qRT-PCR to validate the repeatability of gene expression obtained by RNA-seq. Independent RNA with three replicates of algal cells collected from control group and phosphorous stress was prepared for qRT-PCR analysis. RNA extraction and quality control were performed as described above. Gene specific primers were designed based on the sequences of the eight genes, as shown in Table 3. The relative expression of these genes was quantified by the delta-delta Ct method^37^, which represented the Ct difference between the target gene product and the reference 18rRNA.

**Table 3:** Genes and primers for qRT-PCR detection.

Gene name	Premiers	Primer sequences
Acid phosphatase	AP-F AP-R	TGAAGCCGCCGAAGTACAGTCCTCT GCCACCAGCACAATGGGCACAAAGC
Phosphoglycerate mutase	PM-F PM-R	AGGAGACGGCTGATAATGCTGACGA AGAGTGACCCTGGGTGGACTGTTGT
Sucrose phosphatase	SP-F SP-R	CAGGTCAATGGCGACTCTGGGAATG GCACAGGGTAGTTCAGCCATGTGGA
Glucose-6-phosphate dehydrogenase	G-6-P-F G-6-P-R	CCTAAGGGATGACTGGGACTGTAAA GAATAAGGCGAAGAGTGCTGGAAAC
Oxidoreductase	OD-F OD-R	GGCGACAACCACGCTGGCGAAGAAG GCCGTCAAGTGTCATTTGGCCGTCT
Pyruvate kinase	PK-F PK-R	CCAGCAGTTCACTGCCAAGTCCAGA TGCCTTCCTCAACAGAGTGCCCTTC
Glutamate dehydrogenase	GD-F GD-R	CTATGCTCTGGAGTTGCCTTGCTTC TTCACCTCACCGTTGTCCATTTGTA
Metallo-dependent phosphatase	MP-F MP-R	ATGCCCTGGGATATTCCTTCCTACT CCAAAAGCCAGAAATGTGAAAGTGT
18S rRNA	18S-F 18S-R	TGATAACTTCACGAATCGCACAGCC ATCGAACCCTAATCCTCCGTCACCC

### Statistical analysis

One-way analysis of variance (ANOVA) was applied to analyze the experimental data and the differences were considered significant at *P* < 0.05. In this study, all the treatments were repeated three times and data were reported as the mean ± SD values.

## Conclusions

A high lipid production was obtained with an addition of 2 mgL^−1^ NaH_2_PO_4_.2H_2_O every 2 days in *Scenedesmus* sp. accompanied by carbohydrate degradation. It is probably that starch acted as a primary carbon and energy storage compound, whereas lipids served as long-term, which became major energy storage under stress. At the transcript level, the increase in lipid accumulation was attributed mainly to the decrease of carbohydrate synthesis, enhancement of carbohydrate metabolism, and over-expression of DGAT and KAS under phosphorus stress. Additionally, some genes that encoded key enzymes such as KAS, starch synthase and DGAT were identified. These results might provide a new clue to understand the mechanism of lipid biosynthesis in microalgae.

## Electronic supplementary material

Supplementary Information
