# Supplementary material for: Transcriptome analysis for phosphorus starvation-induced lipid accumulation in Scenedesmus sp
Source: Sci Rep. 2018 Nov 6;8:16420. doi: 10.1038/s41598-018-34650-x (PMC6219579; doi:10.1038/s41598-018-34650-x)
Supplement: Supplementary file 1 — Supplementary Information [file 41598_2018_34650_MOESM1_ESM.pdf]

Supplementary Information

Transcriptome analysis for phosphorus starvation-induced lipid accumulation in *Scenedesmus* sp.

Fangfang Yang, Wenzhou Xiang, Tao Li and Lijuan Long*

Key Laboratory of Tropical Marine Bio-resources and Ecology, South China Sea Institute of Oceanology, Chinese Academy of Sciences, Guangzhou 510301, China

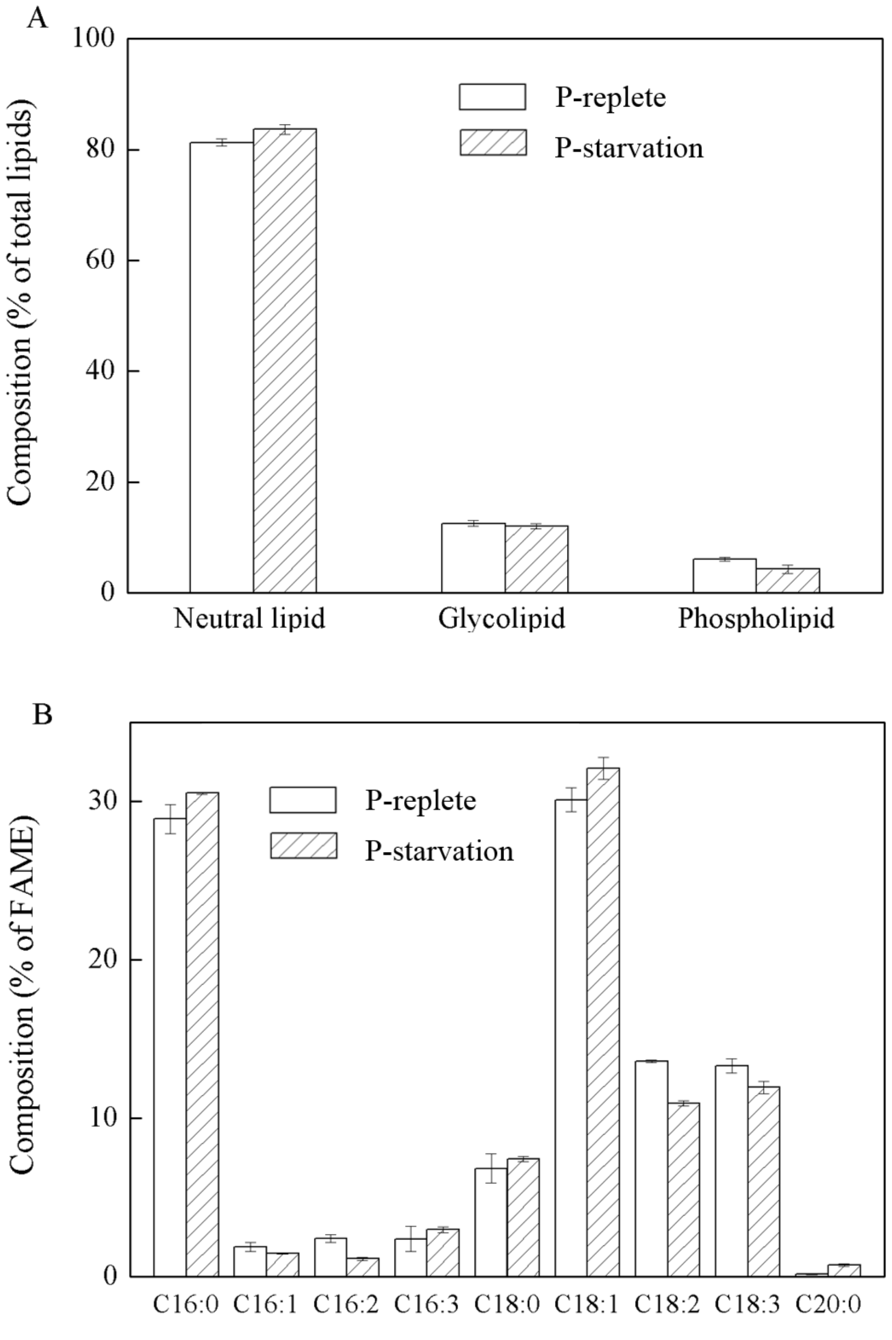

Supplementary Figure S1 (A) Lipid class and (B) fatty acid composition of *Scenedesmus* sp. under phosphorus feeding intervals of 48 h.

Database	No. of unigene hits	Percentage
NR	20356	50.92%
NT	4521	11.31%
Swiss-Prot	10663	26.67%
KEGG	12476	31.21%
COG	10157	25.41%
GO	9265	23.18%
ALL	20983	52.49%

Supplementary Table S1 Summary statistics of functional annotation of unigenes in databases.

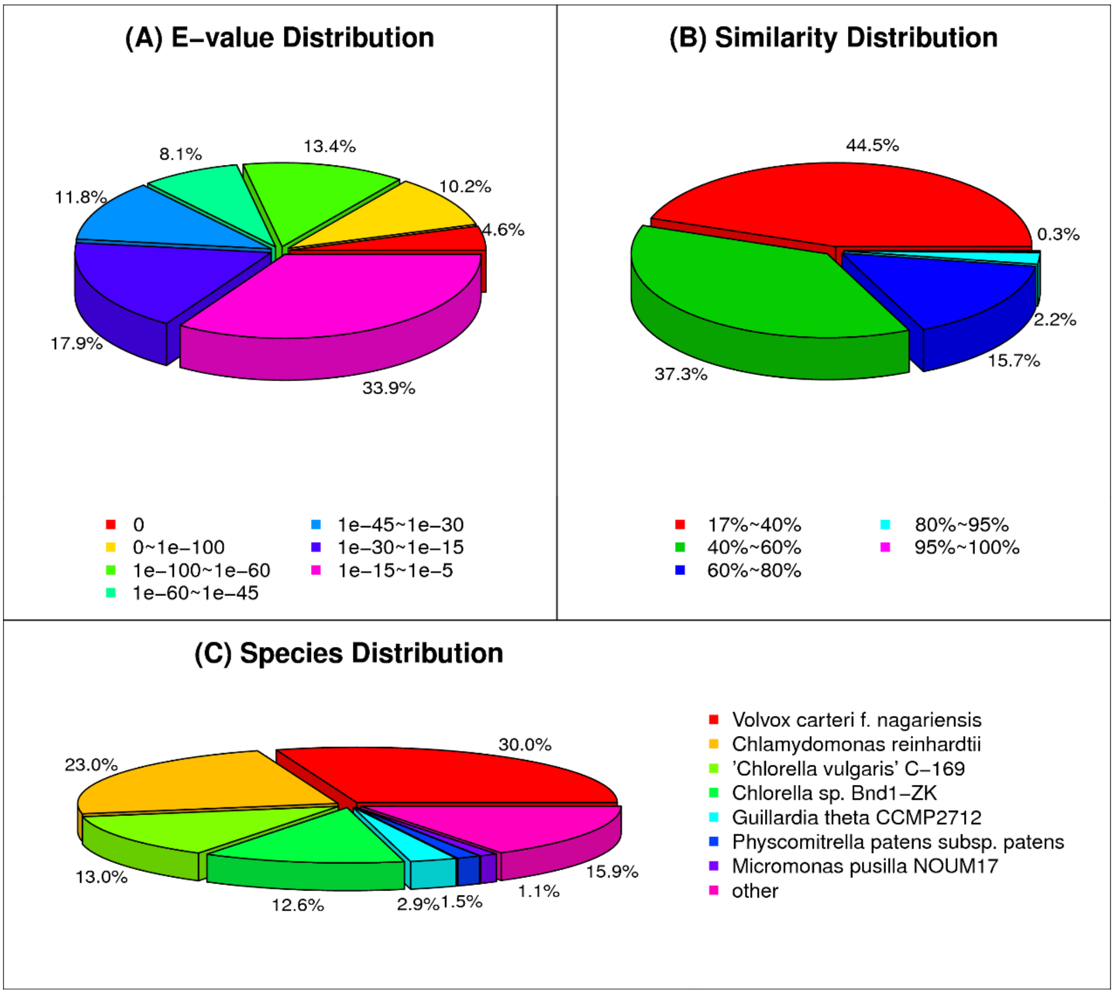

Supplementary Figure S2 Nr classifications. (A) E-value distributions (B) Similarity distributions (C) Species distributions.

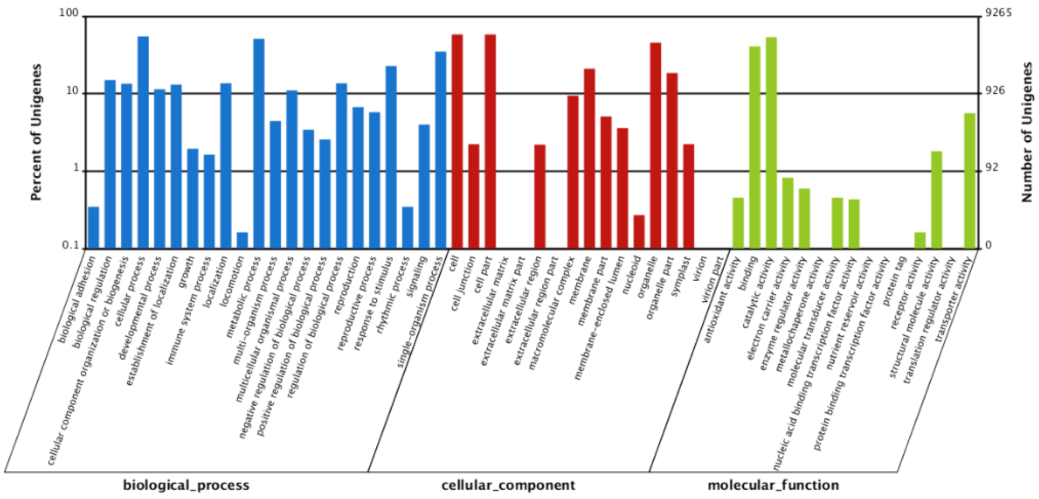

Supplementary Figure S3 GO classification of all unigenes.

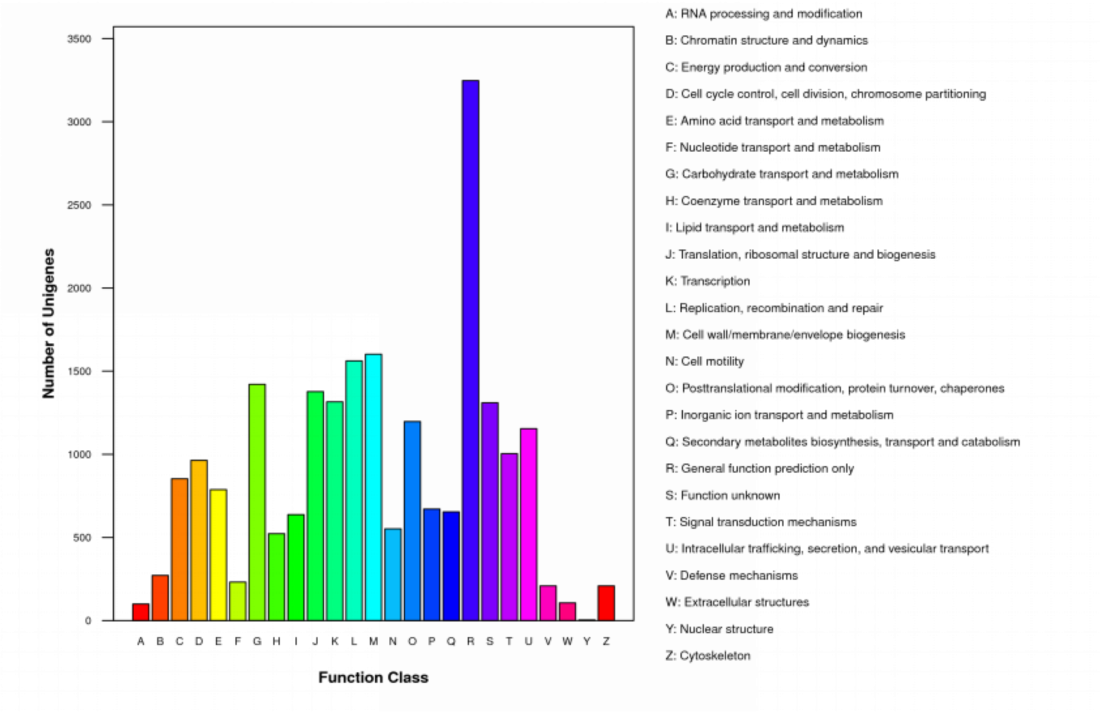

Supplementary Figure S4 COG function classification of all unigenes.

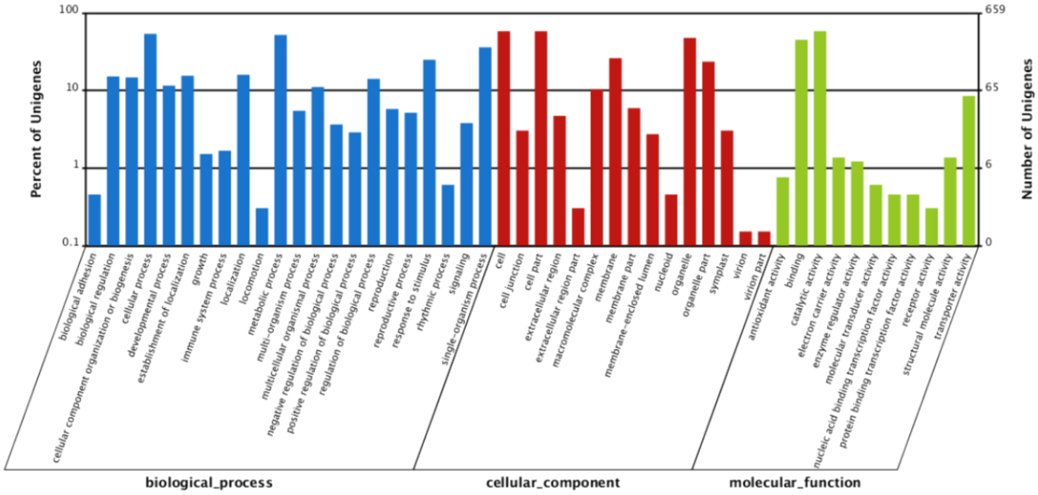

Supplementary Figure S5 GO classification of differentially expressed genes.

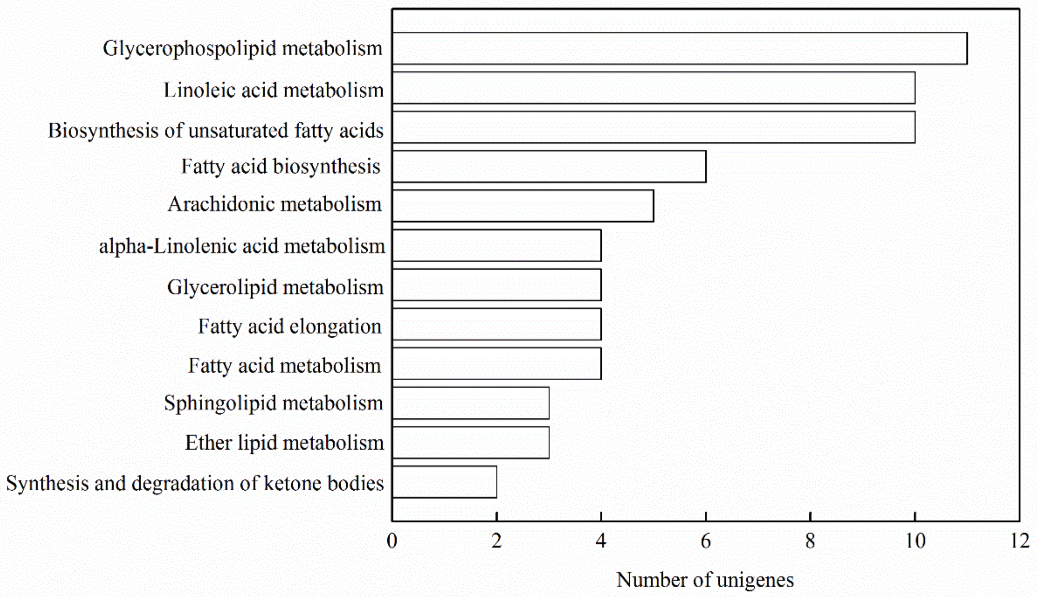

Supplementary Figure S6 Number of differentially expressed genes in the lipid metabolism pathway.
